# The structural characteristics of cellular phospholipid acyl chains required for ABCA1-mediated HDL formation

**DOI:** 10.1016/j.jbc.2025.110457

**Published:** 2025-07-04

**Authors:** Kohjiro Nagao, Mayu Matsuo, Yoshie Hori, Norihiro Namba, Hiroyuki Saito

**Affiliations:** Laboratory of Biophysical Chemistry, Kyoto Pharmaceutical University, Kyoto, Japan

**Keywords:** ABCA1, apolipoprotein A-I, HDL, cholesterol, phospholipid, monounsaturated fatty acid, Δ9-desaturase, SCD1

## Abstract

ATP-binding cassette protein A1 (ABCA1) mediates high-density lipoprotein (HDL) formation by transporting cellular cholesterol and phospholipids to apolipoprotein A-I (apoA-I). Although phospholipids serve as transport substrates for ABCA1, and the membrane constituents surrounding ABCA1, their roles in HDL formation remain unclear. Here, we elucidated the effect of the acyl chain structure of cellular phospholipids on HDL formation, particularly focusing on monounsaturated fatty acid (MUFA)-containing phosphatidylcholine (PC), the predominant phospholipid in most animal cells. PC molecules effluxed to apoA-I had an acyl chain composition similar to cellular PC, both being enriched in MUFA-containing species. Furthermore, manipulating the acyl chain composition of cellular PC by stealoyl-CoA desaturase inhibition or fatty acid supplementation led to similar changes in effluxed PC molecule composition. Thus, ABCA1 can transport various cellular PC molecules, including MUFA-containing species, without apparent preference for their acyl chain structure. Conversely, an appropriate acyl chain composition of cellular phospholipids is required for ABCA1 functional expression. Reducing MUFA content in the cellular phospholipids suppressed ABCA1 expression through two independent mechanisms: first, by inducing an endoplasmic reticulum (ER) stress response that decreases ABCA1 protein production; and second, by causing a folding defect in the ABCA1 protein, leading to immature glycosylation and failure of plasma membrane localization. Excess MUFA supply decreased ABCA1 expression without causing ER stress or defects in glycosylation and localization of ABCA1. Collectively, we revealed the contribution of MUFA-containing PC to HDL formation and identified the structural characteristics of cellular phospholipids required for their transport to apoA-I and functional expression of ABCA1.

The ATP-binding cassette protein A1 (ABCA1) mediates high-density lipoprotein (HDL) formation by transporting cellular cholesterol and phospholipids to apolipoprotein A-I (apoA-I) (1). Since HDL formation by ABCA1 is critical for the removal of excess cholesterol from peripheral cells, a defect in ABCA1 causes Tangier disease, in which patients have a near absence of circulating HDL, prominent cholesterol ester accumulation in tissue macrophages, and premature atherosclerotic vascular disease (2, 3, 4). Therefore, the physiological significance of ABCA1-mediated HDL formation is thought to be excretion of excess cholesterol from cells. However, efflux of cellular phospholipids to apoA-I is also important for HDL formation. A substantial number of cellular phospholipids are transferred to apoA-I during HDL formation (5, 6). Furthermore, phospholipid transport is an essential function of ABCA1 (7, 8, 9). However, despite its importance in HDL formation, the molecular mechanism underlying apoA-I-dependent phospholipid efflux from ABCA1-expressing cells remains unclear.

Glycerophospholipids, which are typical cellular phospholipids, have a structure in which a phosphate-containing polar head group and two strands of fatty acids are attached to a glycerol backbone (10). Owing to the structural diversity of the polar head group and fatty acyl chains, cells can produce a variety of phospholipid molecules. Based on the number of double bonds in the hydrocarbon chain, the fatty acids constituting cellular phospholipids can be classified into three groups: saturated fatty acids (SFAs), which have no double bonds; monounsaturated fatty acids (MUFAs), which have one double bond; and polyunsaturated fatty acids (PUFAs), which have two or more double bonds. These fatty acids are synthesized intracellularly or taken up from outside the cell. In general, PUFAs such as arachidonic acid (C20:4) and docosahexaenoic acid (C22:6) cannot be *de novo* synthesized in mammalian cells (11). In contrast, most mammalian cells can produce SFAs such as palmitic acid (C16:0) and stearic acid (C18:0) from acetyl-CoA and convert SFAs to MUFAs such as palmitoleic acid (C16:1) and oleic acid (C18:1) by introducing a *cis*-double bond (12). The introduction of a *cis*-double at the Δ9-position of the hydrocarbon chain of acyl-CoA is carried out by stearoyl-CoA desaturase 1 (SCD1), an endoplasmic reticulum (ER) membrane protein, which plays a central role in controlling the MUFA content in the acyl chains of cellular phospholipids (13). The *cis*-double bond at the Δ9-position forms a sharp kink in the middle of the phospholipid acyl chains, which affects the molecular behavior of phospholipids and their interactions with neighboring membrane constituents (14). Therefore, the production of MUFA-containing phospholipids has a significant effect on the structure and function of cell membranes.

The most abundant phospholipid transferred from ABCA1-expressing cells to apoA-I is phosphatidylcholine (PC) (5, 15), which contains phosphocholine as a constituent of its polar head group. Like other phospholipids, the acyl chains of cellular PC molecules are composed of various fatty acids (11). Macrophages and fibroblasts expressing ABCA1 preferentially transfer MUFA-containing PC molecules to apoA-I (16). In contrast, ABCA1-expressing HEK293 cells prefer shorter and less unsaturated fatty acid-containing PC molecules as the transport substrate (17). Thus, PC molecules effluxed from ABCA1-expressing cells may possess structural features. However, the ABCA1 protein exhibits similar levels of ATPase activity when reconstituted into PC liposomes with different fatty acyl chains (18), implying that differences in the fatty acyl chain structure of phospholipids do not affect the transport activity of ABCA1. Thus, it remains unclear whether ABCA1 selectively transports PC molecules with specific fatty acyl chains during HDL formation.

The addition of unsaturated fatty acids to the culture medium of ABCA1-expressing cells is reported to reduce the cholesterol efflux to apoA-I by enhancing the degradation of ABCA1 protein through the phosphorylation by PKCδ (19, 20, 21). Overexpression of SCD1 suppresses cholesterol efflux to apoA-I from ABCA1-expressing cells (22). These reports suggest that HDL formation by ABCA1-expressing cells is suppressed by an excess of unsaturated fatty acids in the cellular phospholipid acyl chains. Since the supply of unsaturated fatty acids, including MUFAs, to peripheral cells can increase under conditions such as diabetes and cardiovascular diseases (23, 24, 25), the reduction in HDL formation in the presence of excess amounts of unsaturated fatty acids is likely to have pathological significance. However, the role of physiological levels of unsaturated fatty acids in HDL formation remains unclear. In particular, the requirement for MUFA-containing PC, the predominant phospholipid in most animal cells, for ABCA1-mediated HDL formation is poorly understood.

In this study, we elucidated the effect of the acyl chain structure of cellular phospholipids on HDL formation, particularly focusing on MUFA-containing PC species. In the course of presenting our results, we discuss the structural characteristics of the cellular phospholipids required for their transport to apoA-I and the functional expression of ABCA1.

## Results

### Effect of SCD1 inhibition on membrane lipid composition in BHK/ABCA1 cells

**Figure 1:**
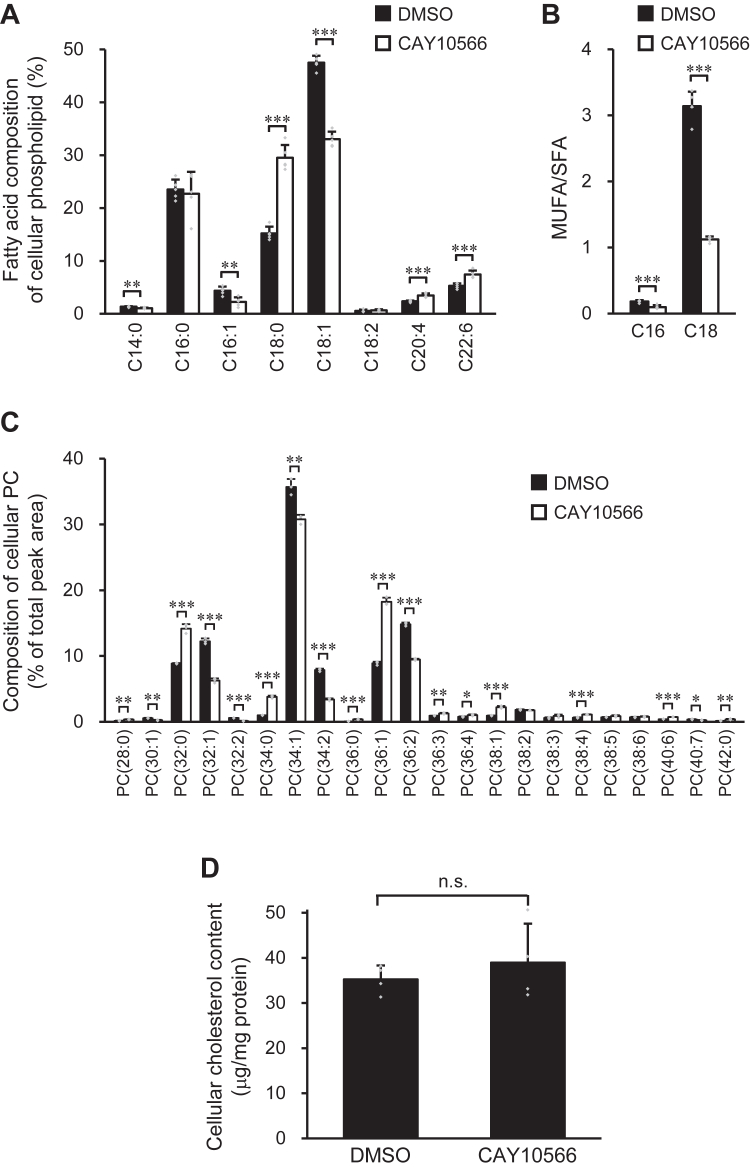
**Effects of SCD1 inhibition on cellular lipid composition.** BHK/ABCA1 cells were treated with 10 nM mifepristone in the presence or absence of 1 μM CAY10566 for 24 h. *A*, *B*, fatty acid composition of cellular phospholipids and MUFA/SFA ratio in cellular phospholipids were analyzed by GC-FID. *C*, composition of cellular PC molecules was analyzed by LC-ESI-MS. PC molecules were presented in the format PC(X:Y), where X denotes the total number of acyl chain carbons and Y denotes the total number of double bonds in acyl chains. *D*, cellular cholesterol content was analyzed by a fluorescent enzyme assay. Mean ± SD (*A*, *B*, *n* = 5; *C*, *n* = 3; *D*, *n* = 4). ∗*p* < 0.05; ∗∗*p* < 0.01; ∗∗∗*p* < 0.001; n.s., not significant.

To elucidate the effect of the acyl chain structure of cellular phospholipids on HDL formation, we manipulated the activity of SCD1, which is crucial for the production of MUFA-containing phospholipids. First, we analyzed the effect of a specific SCD1 inhibitor, CAY10566, on the fatty acid composition of cellular phospholipids. When BHK/ABCA1 cells were treated with the SCD1 inhibitor for 24 h, the proportion of C18:0 in the acyl chains of cellular phospholipids increased by 94%, whereas the proportions of C16:1 and C18:1 decreased by 48% and 31%, respectively (Fig. 1*A*). The reduction in the MUFA/SFA ratio of C18 fatty acids by SCD1 inhibition was more significant than that of C16 fatty acids, indicating that the primary role of SCD1 is the production of C18:1 in BHK/ABCA1 cells (Fig. 1*B*).

Next, we analyzed the molecular composition of cellular PC using liquid chromatography-electrospray ionization-mass spectrometry (LC-ESI-MS) (Fig. 1*C*). The proportions of PC molecules with two double bonds [PC(32:2), PC(34:2), and PC(36:2)] decreased upon SCD1 inhibition. Product ion scan analysis was performed to determine the fatty acid composition of each phospholipid (Fig. S1). Most PC molecules with two double bonds are composed of two MUFAs, such as C16:1 and C18:1 (Fig. S1, *F*–*H*). Thus, it is apparent that a decrease in the level of PC molecules with two double bonds is caused by a reduction in the MUFA content in the phospholipid acyl chains. In contrast, the proportion of PC molecules with no double bonds [PC(32:0) and PC(34:0)] increased after treatment with the SCD1 inhibitor (Figs. 1*C* and S1, *A* and *B*). Since SCD1 inhibition drastically increased the content of C18:0 but not C16:0 in phospholipid acyl chains (Fig. 1*A*), the effect of SCD1 inhibition on the content of PC molecules with one double bond varied depending on the chain length of the SFA constituting these PC molecules. The proportions of PC(32:1) and PC(34:1), which contain C16:0, decreased after SCD1 inhibition (Figs. 1*C* and S1, *C* and *D*). In contrast, the proportion of PC(36:1), which contains C18:0, was increased upon SCD1 inhibition (Figs. 1*C* and S1*E*). These results demonstrated that the inhibition of SCD1 reduced PC molecules rich in MUFAs and increased the content of SFAs, particularly C18:0, in PC acyl chains.

In contrast to the striking effect of SCD1 inhibition on phospholipids, the cellular cholesterol content was not altered by SCD1 inhibition (Fig. 1*D*). Furthermore, the inhibition of SCD1 did not induce cytotoxicity as assessed by lactate dehydrogenase (LDH) release (Fig. S2). These results demonstrate that SCD1 inhibition specifically reduces the MUFA content in the phospholipid acyl chains of BHK/ABCA1 cells.

### Effect of SCD1 inhibition on lipid efflux to apoA-I from ABCA1-expressing cells

**Figure 2:**
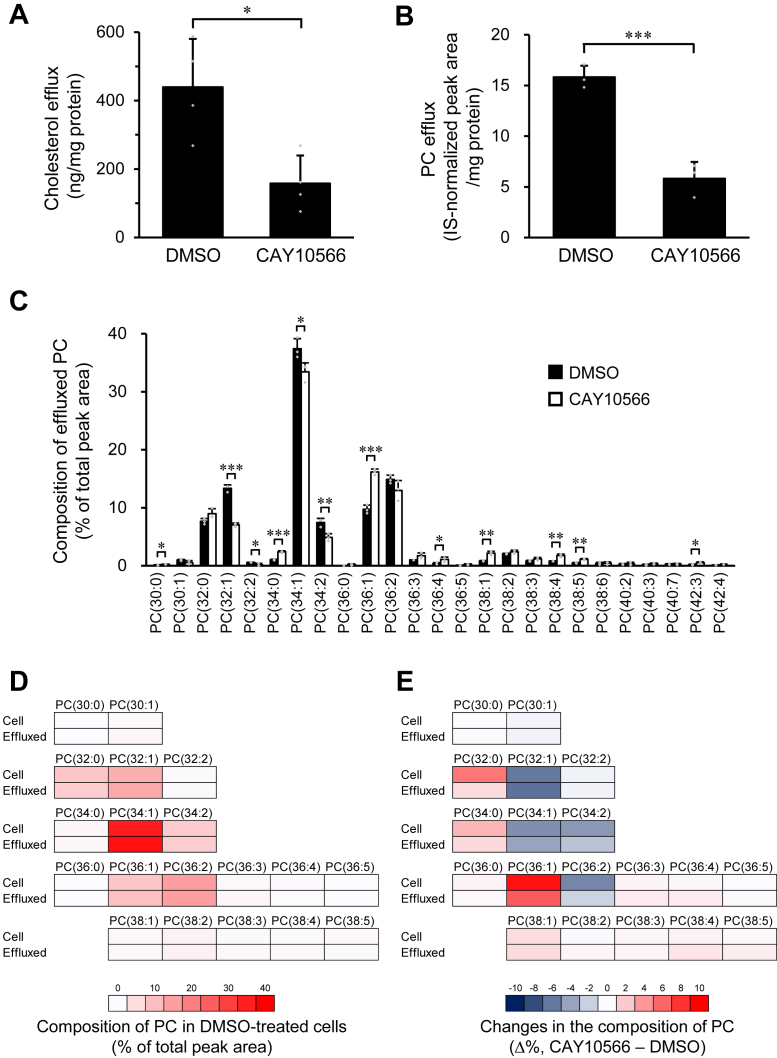
**Effects of SCD1 inhibition on cellular lipid efflux.** BHK/ABCA1 cells were treated with 10 nM mifepristone in the presence or absence of 1 μM CAY10566 for 24 h, and then incubated with 10 μg/ml apoA-I for 4 h. ApoA-I-dependent efflux of cholesterol (*A*) and PC (*B*) was analyzed by a fluorescent enzyme assay and LC-ESI-MS, respectively. *C*, composition of PC molecules effluxed to apoA-I was analyzed by LC-ESI-MS. PC molecules were presented in the format PC(X:Y), where X denotes the total number of acyl chain carbons and Y denotes the total number of double bonds in acyl chains. *D*, composition of cellular PC and apoA-I-dependently effluxed PC in DMSO-treated cells. *E*, changes in the composition of cellular PC and apoA-I-dependently effluxed PC caused by CAY10566 treatment. *D*, *E*, the heat map was drawn using the values in Figures 1*C* and 2*C*. Mean ± SD (*A*, *n* = 4; *B*, *C*, *n* = 3). ∗*p* < 0.05; ∗∗*p* < 0.01; ∗∗∗*p* < 0.001.

Next, we analyzed the effect of SCD1 inhibition on apoA-I-dependent lipid efflux from BHK/ABCA1 cells. As shown in Figure 2*A*, apoA-I-dependent cholesterol efflux decreased by 64% after SCD1 inhibition. Similarly, the efflux of PC to apoA-I was reduced by 63% following SCD1 inhibition (Fig. 2*B*). These results demonstrate that decreasing MUFA content in the acyl chains of cellular phospholipids suppresses ABCA1-mediated lipid efflux to apoA-I.

We then analyzed the fatty acid composition of the PC molecules effluxed to apoA-I using LC-ESI-MS. MUFA-containing PC species, such as PC(32:1), PC(34:1), and PC(36:2), were abundantly effluxed to apoA-I in vehicle-treated BHK/ABCA1 cells (Fig. 2*C*, filled bars). As shown in the heat map (Fig. 2*D*), the composition of PC molecules effluxed to apoA-I from vehicle-treated cells (Fig. 2*C*, filled bars) resembled that of cellular PC in vehicle-treated cells (Fig. 1*C*, filled bars), indicating that the acyl chain composition of PC molecules effluxed to apoA-I reflects the cellular PC composition.

We examined the effect of SCD1 inhibition on the efflux of each PC species. When the amount of PC effluxed to apoA-I was evaluated for each species, most PC species showed decreased efflux to apoA-I upon treatment with the SCD1 inhibitor (Fig. S3). However, the degree of reduction in apoA-I-dependent efflux caused by SCD1 inhibition differed among PC species (Fig. S3), indicating that the composition of PC molecules effluxed to apoA-I was significantly altered by SCD1 inhibition (Fig. 2*C*). In particular, PC(32:1), PC(34:1), and PC(34:2), whose cellular contents decreased in SCD1-inhibited cells (Fig. 1*C*), showed a reduction in their proportions in the PC molecules effluxed to apoA-I under SCD1-inhibited conditions (Fig. 2*C*). In contrast, PC(34:0) and PC(36:1), whose cellular contents increased in SCD1-inhibited cells (Fig. 1*C*), showed an increase in their proportions in the PC molecules effluxed to apoA-I upon SCD1 inhibition (Fig. 2*C*). Thus, the degrees of change in the acyl chain composition of cellular and effluxed PC caused by SCD1 inhibition were comparable (Fig. 2*E*), again indicating that the composition of PC molecules effluxed to apoA-I reflects that of cellular PC molecules.

### Structural characteristics of PC molecules effluxed to apoA-I from ABCA1-expressing cells

**Figure 3:**
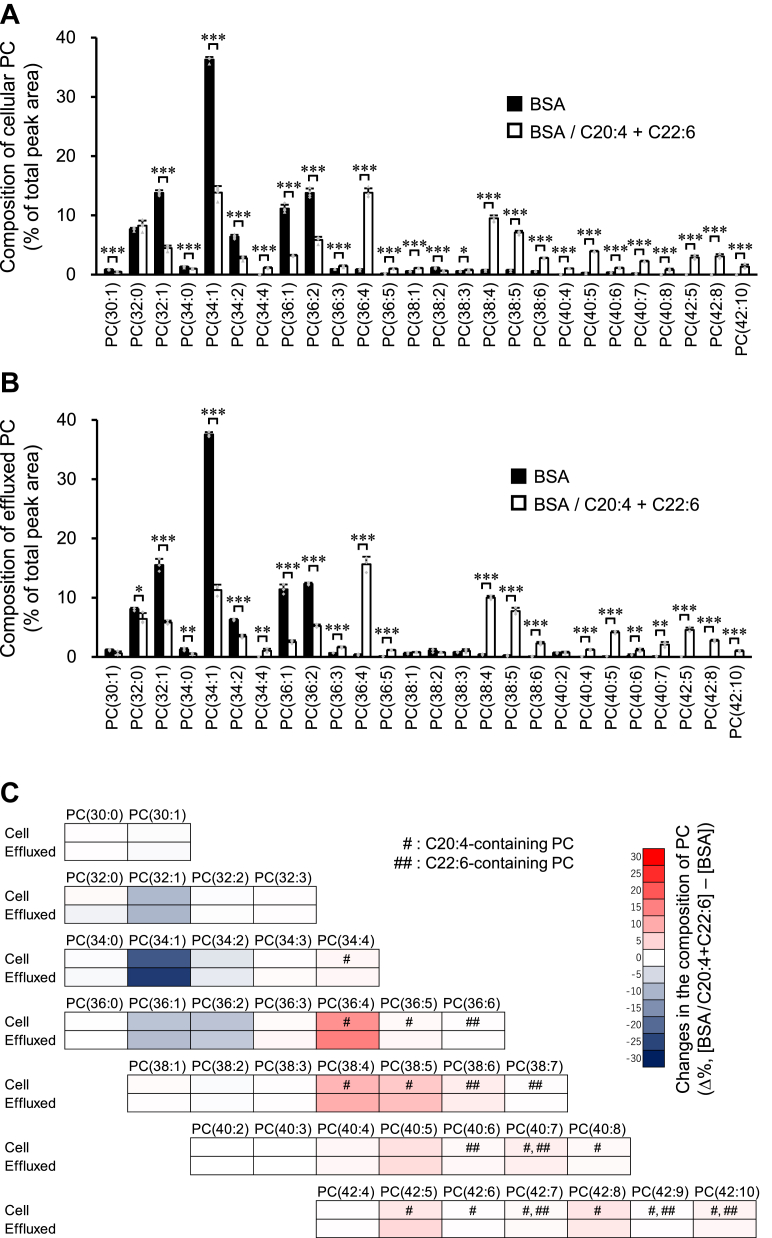
**Effects of PUFA supplementation on PC efflux.** BHK/ABCA1 cells were treated with 10 nM mifepristone in the presence or absence of 50 μM C20:4 and 50 μM C22:6 for 24 h. *A*, composition of cellular PC molecules was analyzed by LC-ESI-MS. PC molecules were presented in the format PC(X:Y), where X denotes the total number of acyl chain carbons and Y denotes the total number of double bonds in acyl chains. *B*, cells were incubated with 10 μg/ml apoA-I for 4 h. Composition of PC molecules effluxed to apoA-I was analyzed by LC-ESI-MS. *C*, changes in the composition of cellular PC and apoA-I-dependently effluxed PC caused by C20:4 and C22:6 supplementation. The heat map was drawn using the values in Figure 3, *A* and *B*. Mean ± SD (*A*, *n* = 4; *B*, *n* = 3). ∗*p* < 0.05; ∗∗*p* < 0.01; ∗∗∗*p* < 0.001.

The fact that the acyl chain composition of PC molecules effluxed to apoA-I was similar to that of cellular PC molecules (Fig. 2, *D* and *E*) raised the possibility that ABCA1 transfers cellular PC molecules to apoA-I regardless of differences in the structure of their fatty acyl chains. To investigate this possibility, we altered the acyl chain composition of cellular PC using a method other than SCD1 inhibition. Under normal culture conditions, the PUFA content in the acyl chains of cellular PC was low in BHK/ABCA1 cells. However, the addition of C20:4 and C22:6 to the culture medium significantly increased the proportion of added PUFAs in the acyl chains of cellular PC (Fig. 3*A*). Due to the increased PUFA content, the proportions of PC species composed exclusively of MUFAs and SFAs, such as PC(32:1), PC(34:1), PC(36:1), PC(34:2), and PC(36:2) in cellular PC were significantly decreased by the addition of C20:4 and C22:6 to the culture medium (Fig. 3*A*). Next, we evaluated the effect of supplementing the culture medium with PUFAs on the acyl chain composition of the PC molecules effluxed to apoA-I. The proportion of PUFA-containing species in the PC molecules effluxed to apoA-I increased with the addition of C20:4 and C22:6 (Fig. 3*B*). In contrast, the proportion of PC species composed exclusively of MUFAs and SFAs in the PC molecules effluxed to apoA-I was reduced by supplementing the culture medium with C20:4 and C22:6 (Fig. 3*B*). Thus, similar to the results observed with the SCD1 inhibitor treatment (Fig. 2*E*), the addition of C20:4 and C22:6 to the culture medium caused comparable changes in the acyl chain composition of both the cellular and effluxed PC molecules (Fig. 3*C*). These results demonstrate that ABCA1 can transport a variety of cellular PC molecules to apoA-I without an apparent preference for their acyl chain structure.

### Effect of SCD1 inhibition on the expression of ABCA1

**Figure 4:**
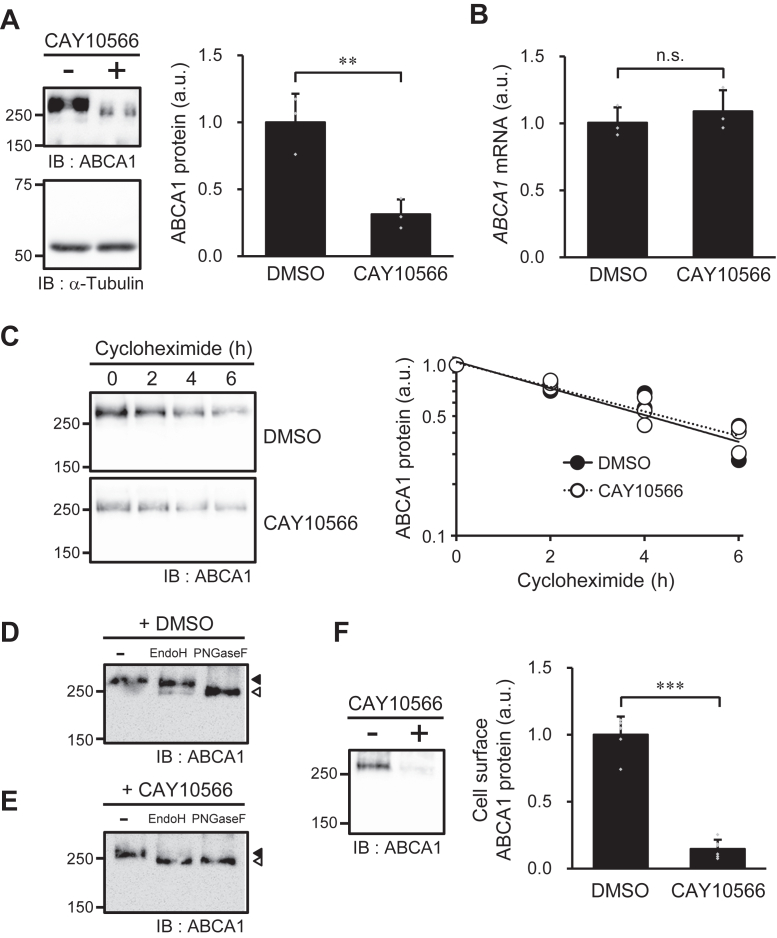
**Effects of SCD1 inhibition on ABCA1 expression.** BHK/ABCA1 cells were treated with 10 nM mifepristone in the presence or absence of 1 μM CAY10566 for 24 h. *A*, the amounts of ABCA1 and α-tubulin protein were detected with specific antibodies. *B*, the amount of *ABCA1* mRNA was determined by RT-PCR. *C*, cells were treated with cycloheximide (100 μg/ml) for 0, 2, 4, and 6 h in the presence or absence of 1 μM CAY10566, and the amounts of ABCA1 protein were detected with specific antibodies (*n* = 3). Glycosylation analysis of ABCA1 in BHK/ABCA1 cells treated with DMSO (*D*) and 1 μM CAY10566 (*E*). Closed arrowhead indicates the position of ABCA1 in sample not treated with glycosidases; open arrowhead indicates the position of deglycosylated ABCA1. *F*, the cells were treated with sulfo-NHS-biotin, and the biotinylated cell surface proteins were precipitated with avidin-agarose and detected by immunoblotting. *A*, *C*, *D*–*F*, numbers on the *left* of the *panels* indicate the molecular weights (kDa) of size markers. Mean ± SD (*A*, *B*, *F*, *n* = 3). ∗∗*p* < 0.01; ∗∗∗*p* < 0.001; n.s., not significant; a.u., arbitrary unit.

To investigate the cause of reduced lipid efflux in SCD1-inhibited cells (Fig. 2, *A* and *B*), we assessed the expression level of ABCA1. As shown in Figure 4*A*, treatment with the SCD1 inhibitor reduced the amount of ABCA1 protein by 68%. In contrast, the level of *ABCA1* mRNA was not affected by treatment with the SCD1 inhibitor (Fig. 4*B*). Furthermore, the degradation rates of the ABCA1 protein were comparable in vehicle- and SCD1 inhibitor-treated cells (Fig. 4*C*). Therefore, SCD1 inhibition appears to reduce ABCA1 expression by suppressing ABCA1 protein production.

In cells treated with the SCD1 inhibitor, not only was the expression of ABCA1 reduced, but the molecular size of ABCA1 was also reduced (Fig. 4*A*). The degree of glycosylation affects the molecular size of ABCA1 (26). ABCA1 undergoes addition of high-mannose-type oligosaccharides in the ER, and the sugar chains of ABCA1 mature into complex-type oligosaccharides during transport through the Golgi apparatus to the plasma membrane (26). Therefore, we evaluated the effect of SCD1 inhibition on the glycosylation process of ABCA1. Consistent with previous reports, the sugar chains of ABCA1 in vehicle-treated cells were resistant to Endoglycosidase H (Endo H), which removes high-mannose-type oligosaccharides but not complex-type oligosaccharides from N-linked glycoproteins (Fig. 4*D*, second lane from the left), and sensitive to peptide N-glycosidase F (PNGase F), which cleaves most of the N-linked sugar chains, including complex-type oligosaccharides (Fig. 4*D*, third lane from the left). Thus, ABCA1 can be glycosylated to form complex-type oligosaccharides in vehicle-treated BHK/ABCA1 cells. In contrast, the sugar chains of ABCA1 in the SCD1 inhibitor-treated cells were sensitive to Endo H (Fig. 4*E*). These results suggest that SCD1 inhibition affects the glycosylation process of ABCA1, leading to the retention of high mannose-type immature oligosaccharides on ABCA1 in SCD1-inhibited cells. Because ABCA1 oligosaccharides undergo maturation during trafficking from the ER to the plasma membrane, we investigated whether inhibition of SCD1 affects the cell surface expression of ABCA1. SCD1 inhibition reduced the expression of ABCA1 on the cell surface by 84% (Fig. 4*F*). These results demonstrated that SCD1 inhibition suppresses the expression of mature ABCA1 protein in the plasma membrane, where HDL formation mainly occurs in BHK/ABCA1 cells (27).

SCD1 inhibition reduced total cellular levels of low-density lipoprotein receptor (LDLR) (Fig. S4*A*); however, LDLR complex-type glycosylation was not affected by SCD1 inhibition (Fig. S4, *B* and *C*). Furthermore, the degree of reduction in LDLR on the cell surface caused by SCD1 inhibition was comparable to the degree of reduction in LDLR in the whole cells (Fig. S4*D*), indicating that SCD1 inhibition did not affect the translocation efficiency of LDLR to the cell surface. Therefore, it is apparent that SCD1 inhibition does not affect the general mechanisms of maturation or intracellular translocation of membrane proteins.

### Involvement of ER stress response in the downregulation of ABCA1 in SCD1-inhibited cells

**Figure 5:**
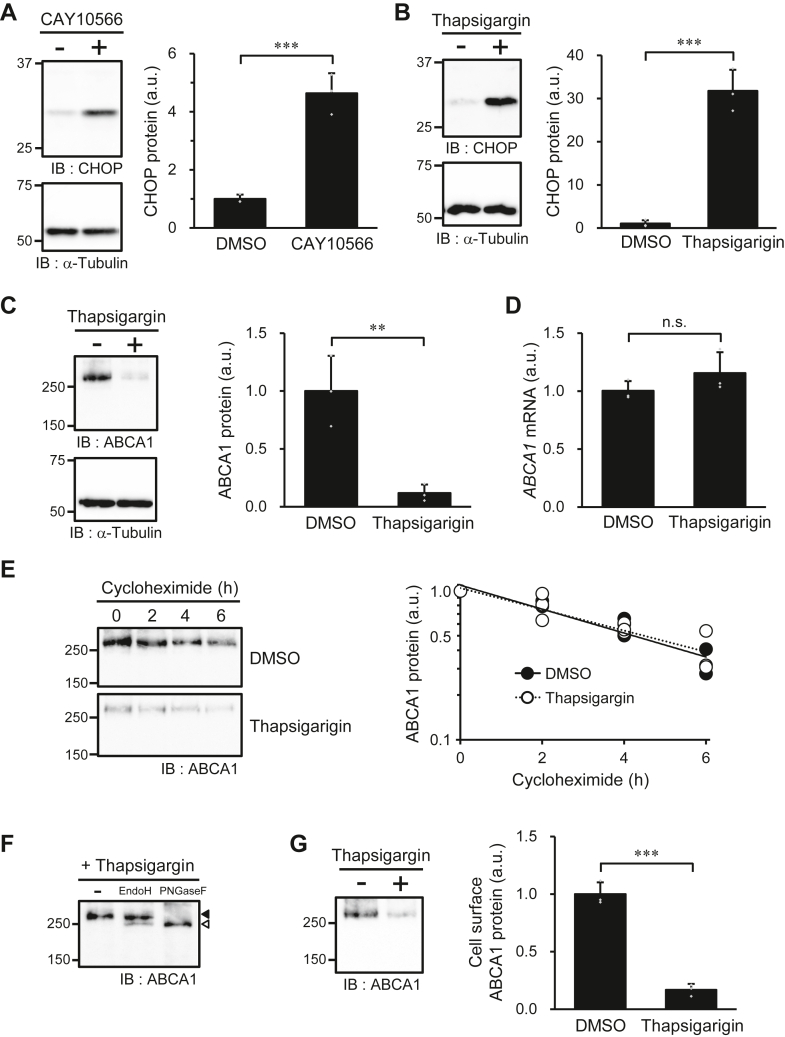
**Effects of ER stress response on ABCA1 expression.** BHK/ABCA1 cells were treated with 10 nM mifepristone in the presence or absence of 1 μM CAY10566 or 1 μM thapsigargin for 24 h. *A* and *B*, the amounts of CHOP and α-tubulin protein were detected with specific antibodies. *C*, the amounts of ABCA1 and α-tubulin protein were detected with specific antibodies. *D*, the amount of *ABCA1* mRNA was determined by RT-PCR. *E*, cells were treated with cycloheximide (100 μg/ml) for 0, 2, 4, and 6 h in the presence or absence of 1 μM thapsigargin, and the amounts of ABCA1 protein were detected with specific antibodies (*n* = 3). *F*, glycosylation analysis of ABCA1 in BHK/ABCA1 cells treated with 1 μM thapsigargin. Closed arrowhead indicates the position of ABCA1 in sample not treated with glycosidases; open arrowhead indicates the position of deglycosylated ABCA1. *G*, the cells were treated with sulfo-NHS-biotin, and the biotinylated cell surface proteins were precipitated with avidin-agarose and detected by immunoblotting. *A*–*C* and *E*–*G*, numbers on the left of the panels indicate the molecular weights (kDa) of size markers. Mean ± SD (*A*–*D*, *G*, *n* = 3). ∗∗*p* < 0.01; ∗∗∗*p* < 0.001; n.s., not significant; a.u., arbitrary unit.

It has been reported that SFA accumulation causes ER stress (28, 29). When ER stress arises, cells attempt to alleviate further strain by suppressing protein translation (30). Since the inhibition of SCD1 appeared to suppress the production of ABCA1 protein (Fig. 4, *A*–*C*), we investigated whether the ER stress response was responsible for the decreased expression of ABCA1 in SCD1-inhibited cells. The expression level of CHOP, an ER stress marker, increased 4.6-fold with SCD1 inhibitor treatment (Fig. 5*A*), indicating that ER stress arose in BHK/ABCA1 cells upon SCD1 inhibition. Similar to the results obtained with SCD1 inhibition, treatment with thapsigargin, an inducer of ER stress, increased the expression level of CHOP 31-fold (Fig. 5*B*) and decreased the amount of ABCA1 protein to 12% of that in vehicle-treated cells (Fig. 5*C*) without affecting the *ABCA1* mRNA level (Fig. 5*D*) or the degradation rate of ABCA1 protein (Fig. 5*E*). Thus, it appears that SCD1 inhibition activates the ER stress response, leading to decreased production of ABCA1 protein.

Although the expression level of ABCA1 was decreased by the induction of ER stress, the molecular size (Fig. 5*C*) and glycosylation (Fig. 5*F*) of ABCA1 were not affected by thapsigargin treatment. Thapsigargin treatment reduced the amount of ABCA1 on the cell surface by 83% (Fig. 5*G*). However, the degree of reduction in the amount of ABCA1 on the cell surface (Fig. 5*G*) and in whole cells (Fig. 5*C*) was comparable, indicating that thapsigargin treatment did not affect the efficiency of ABCA1 translocation to the plasma membrane. These results revealed that immature glycosylation and defects in ABCA1 localization upon SCD1 inhibition are not caused by the ER stress response.

### Effect of SCD1 inhibition on ABCA1 protein folding

**Figure 6:**
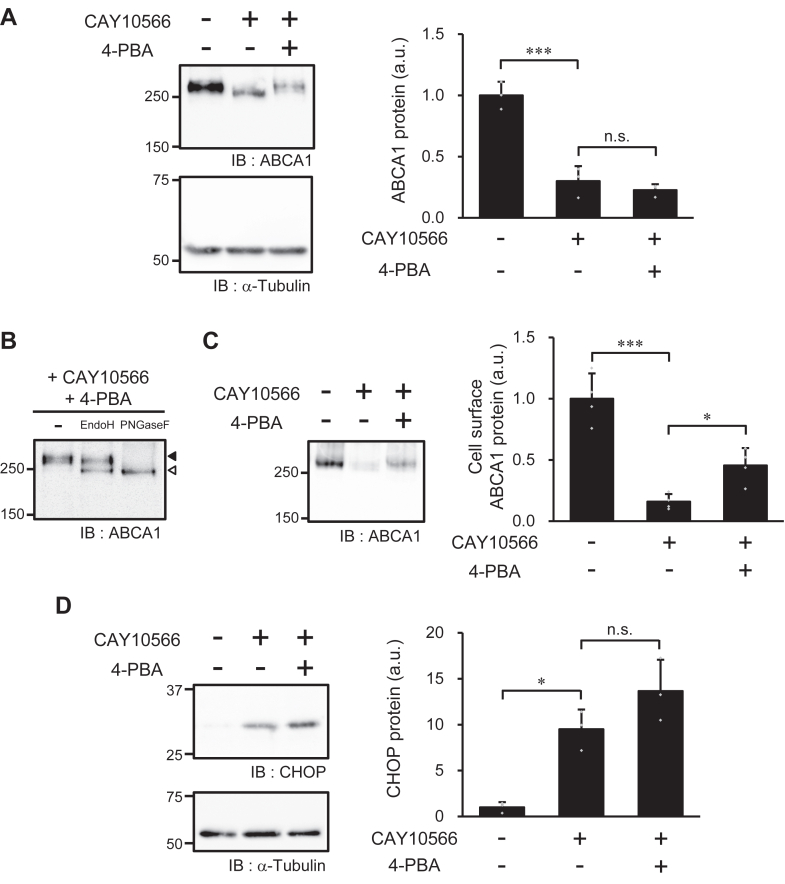
**Effects of chemical chaperon on ABCA1 expression.** BHK/ABCA1 cells were treated with 10 nM mifepristone in the presence or absence of 1 μM CAY10566 and 10 mM 4-PBA for 24 h. *A*, the amounts of ABCA1 and α-tubulin protein were detected with specific antibodies. *B*, glycosylation analysis of ABCA1. Closed arrowhead indicates the position of ABCA1 in sample not treated with glycosidases; open arrowhead indicates the position of deglycosylated ABCA1. *C*, the cells were treated with sulfo-NHS-biotin, and the biotinylated cell surface proteins were precipitated with avidin-agarose and detected by immunoblotting. *D*, the amounts of CHOP and α-tubulin protein were detected with specific antibodies. (*A*–*D*) Numbers on the left of the panels indicate the molecular weights (kDa) of size markers. Mean ± SD (*A*, *C*, *D*, *n* = 3). ∗*p* < 0.05; ∗∗∗*p* < 0.001; n.s., not significant; a.u., arbitrary unit.

Various ABCA1 mutations found in patients with Tangier disease cause misfolding of the ABCA1 protein (31). Misfolded ABCA1 is recognized by a protein quality control system in the ER, which results in the failure of ABCA1 translocation from the ER to the plasma membrane through the Golgi apparatus (26). Thus, these Tangier mutants have defects in protein glycosylation across the ER and Golgi apparatus (32). In addition, the composition of membrane phospholipids affects the structure of various membrane proteins (33, 34). Therefore, we hypothesized that SCD1 inhibition leads to a folding defect in the ABCA1 protein, which causes retention of ABCA1 in the ER and immature glycosylation of ABCA1. Thus, we evaluated whether 4-phenylbutyric acid (4-PBA), a chemical chaperone reported to rescue the folding defect in ABCA1 Tangier mutants (35), could restore the defect in ABCA1 expression in SCD1-inhibited cells. Although the reduced amount of ABCA1 protein was not rescued by 4-PBA (Fig. 6*A*), the reduced size of ABCA1 (Fig. 6*A*) and the defects in ABCA1 glycosylation (Fig. 6*B*) and cell surface localization (Fig. 6*C*) were partially restored by the addition of 4-PBA at a concentration reported to rescue ABCA1 Tangier mutants (35). As 4-PBA did not reduce the expression of CHOP in SCD1-inhibited cells (Fig. 6*D*), the effect of 4-PBA on the glycosylation and localization of ABCA1 was not mediated by the suppression of ER stress. These results suggest that SCD1 inhibition causes a folding defect in ABCA1, leading to immature glycosylation and failure of ABCA1 localization to the plasma membrane.

### Effect of excess MUFA on ABCA1-mediated HDL formation

**Figure 7:**
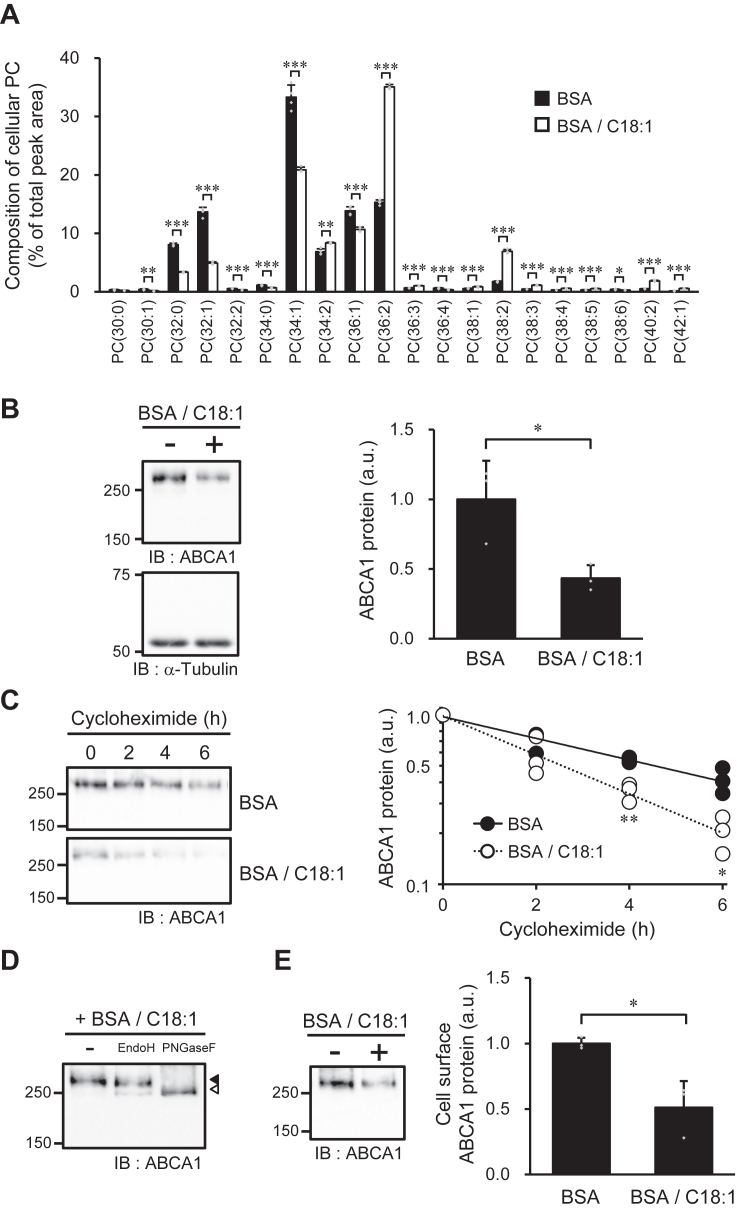
**Effects of excess MUFA on ABCA1 expression.** BHK/ABCA1 cells were treated with 10 nM mifepristone in the presence or absence of 125 μM C18:1 for 24 h. *A*, composition of cellular PC molecules was analyzed by LC-ESI-MS. PC molecules were presented in the format PC(X:Y), where X denotes the total number of acyl chain carbons and Y denotes the total number of double bonds in acyl chains. *B*, the amounts of ABCA1 and α-tubulin protein were detected with specific antibodies. *C*, cells were treated with cycloheximide (100 μg/ml) for 0, 2, 4, and 6 h in the presence or absence of 125 μM C18:1, and the amounts of ABCA1 protein were detected with specific antibodies (*n* = 3). *D*, glycosylation analysis of ABCA1. Closed arrowhead indicates the position of ABCA1 in sample not treated with glycosidases; open arrowhead indicates the position of deglycosylated ABCA1. *E*, the cells were treated with sulfo-NHS-biotin, and the biotinylated cell surface proteins were precipitated with avidin-agarose and detected by immunoblotting. *B*–*E*, numbers on the *left* of the panels indicate the molecular weights (kDa) of size markers. Mean ± SD (*A*, *n* = 4; *B*, *E*, *n* = 3). ∗*p* < 0.05; ∗∗*p* < 0.01; ∗∗∗*p* < 0.001; a.u., arbitrary unit.

Finally, the effect of increasing the cellular MUFA content on ABCA1 was evaluated. The proportion of C18:1-containing species, such as PC(34:2), PC(36:2), and PC(38:2), in cellular PC increased with the addition of C18:1 to the culture medium (Fig. 7*A*). Consistent with previous reports (19, 20), the addition of C18:1 significantly reduced ABCA1 levels (Fig. 7*B*). Furthermore, the half-life of ABCA1 protein was shortened from 4.5 h to 2.6 h by C18:1 treatment (Fig. 7*C*), demonstrating that the addition of C18:1 enhanced the degradation of ABCA1 protein.

Addition of C18:1 to the culture medium significantly increased cellular triacylglycerol content in BHK/ABCA1 cells (Fig. S5*A*). Reflecting the requirement for DGAT1 in triacylglycerol synthesis, the accumulation of triacylglycerol in the presence of excess C18:1 was suppressed by treatment with the DGAT1 inhibitor T863 (Fig. S5*A*). Since the addition of C18:1 reduced ABCA1 expression levels, even in the presence of a DGAT1 inhibitor (Fig. S5*B*), likely, triacylglycerol accumulation is not necessary to reduce ABCA1 expression under conditions of excess C18:1.

Addition of C18:1 did not affect the molecular size of ABCA1 (Fig. 7*B*) or its modification with complex-type oligosaccharides (Fig. 7*D*). Although C18:1 treatment reduced the expression level of ABCA1 on the cell surface by 48% (Fig. 7*E*), this reduction was comparable to the decrease observed in the expression level of ABCA1 in whole cells (Fig. 7*B*), suggesting that C18:1 treatment did not alter the distribution of ABCA1 between the cell surface and within cells. Thus, it is apparent that excess C18:1 does not suppress the maturation of the ABCA1 protein. Since the addition of C18:1 did not increase but rather decreased CHOP expression (Fig. S6), the decrease in ABCA1 expression in the C18:1-treated cells was not due to ER stress. Thus, both an excess of MUFA supply and a reduction in cellular MUFAs lead to decreased ABCA1 expression through different mechanisms.

We assessed whether endogenous ABCA1 expression is also regulated by the MUFA content in cellular phospholipids. In murine Raw264 macrophages, SCD1 inhibition reduced ABCA1 protein levels (Fig. S7*A*). SCD1 inhibition induced immature glycosylation of ABCA1 (Fig. S7, *E* and *F*) and ER stress, which suppressed ABCA1 expression in Raw264 cells (Fig. S7, *B*–*D*). Furthermore, the addition of C18:1 to the culture medium of Raw264 cells reduced the expression level of ABCA1 (Fig. S8*A*) and promoted ABCA1 protein degradation (Fig. S8*B*). Thus, endogenous ABCA1 expression could also be regulated by cellular MUFA content.

We attempted to increase the cellular MUFA content using methods other than the addition of C18:1. Consistent with the results of the previous report (22), the exogenous expression of SCD1 did not affect ABCA1 expression levels (Fig. S9*A*). However, transient expression of SCD1 did not significantly alter the fatty acid composition of cellular PC molecules in BHK/ABCA1 cells (Fig. S9*B*). This may be attributed to the intrinsically high levels of SCD1-mediated C18:1 production in BHK cells (Fig. 1*B*) or the dynamic transcriptional and post-translational regulation of SCD1 expression (36, 37, 38). Thus, an increased supply of MUFAs from outside the cell, as observed in conditions such as diabetes and cardiovascular disease, may be required to suppress ABCA1 expression due to excess MUFAs.

**Figure 8:**
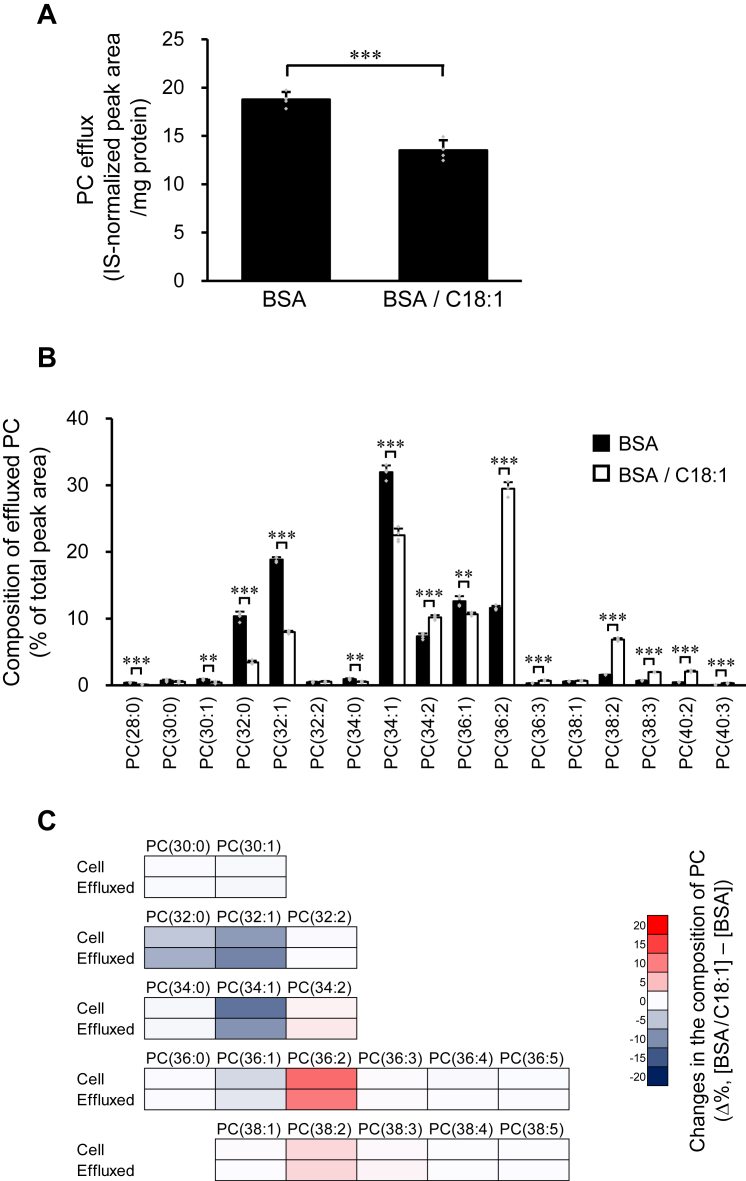
**Effects of excess MUFA on PC efflux.** BHK/ABCA1 cells were treated with 10 nM mifepristone in the presence or absence of 125 μM C18:1 for 24 h, and then incubated with 10 μg/ml apoA-I for 4 h. *A*, apoA-I-dependent efflux of PC was analyzed by LC-ESI-MS. *B*, composition of PC molecules effluxed to apoA-I was analyzed by LC-ESI-MS. PC molecules were presented in the format PC(X:Y), where X denotes the total number of acyl chain carbons and Y denotes the total number of double bonds in acyl chains. *C*, changes in the composition of cellular PC and apoA-I-dependently effluxed PC caused by C18:1 supplementation. The heat map was drawn using the values in Figures 7*A* and 8*B*. Mean ± SD (*A*, *B*, *n* = 3). ∗∗*p* < 0.01; ∗∗∗*p* < 0.001.

Reflecting the reduction in ABCA1 expression, the total PC efflux to apoA-I was reduced by the addition of C18:1 (Fig. 8*A*). However, the proportion of C18:1-containing species in the PC molecules effluxed to apoA-I increased with the addition of C18:1 (Fig. 8*B*). Similar to the results of SCD1 inhibition and PUFA supplementation (Figs. 2*E* and 3*C*), the addition of C18:1 to the culture medium caused comparable changes in the acyl chain compositions of both cellular and effluxed PC molecules (Fig. 8*C*). Therefore, increasing the cellular content of MUFA-containing PC species did not result in them being transported more efficiently than other PC species.

These results revealed that MUFA-containing PC molecules, which are abundant in most animal cells, are effluxed to apoA-I from ABCA1-expressing cells in large quantities, but are not preferentially transported to apoA-I over other PC species. Furthermore, appropriate MUFA content in the acyl chains of cellular phospholipids is required for the functional expression of ABCA1.

## Discussion

PC is the most abundant phospholipid transferred to apoA-I from ABCA1-expressing cells and is a major constituent of the cell membrane in which ABCA1, a membrane protein with 12 transmembrane helices, is embedded (5, 10, 15). The structure of membrane PC molecules affects the physicochemical properties of the bilayer membrane and the functions of a wide range of membrane proteins (11, 33, 39). Therefore, a close functional interaction is expected between ABCA1 and cellular PC molecules. However, the effect of the chemical structure of cellular PC molecules on HDL formation remains unclear. In this study, we attempted to reveal the structural characteristics of the cellular phospholipids required for their transport to apoA-I and the functional expression of ABCA1.

The HDL particles produced by BHK/ABCA1 cells were rich in MUFA-containing PC (Fig. 2*C*, filled bars). However, when cellular MUFA content was reduced by SCD1 inhibition, the proportion of MUFA-containing species in the PC molecules effluxed to apoA-I decreased (Fig. 2*C*). Furthermore, reducing cellular PC species composed exclusively of MUFAs and SFAs by adding C20:4 and C22:6 to the culture medium decreased the proportion of these PC species in the PC molecules effluxed to apoA-I (Fig. 3*B*). Conversely, when the cellular C18:1 content was increased by adding C18:1 to the culture medium, the proportion of C18:1-containing species in the PC molecules effluxed to apoA-I increased (Fig. 8*B*). Furthermore, these interventions of acyl chain composition of cellular phospholipids affected apoA-I-dependent efflux of a wide range of species, not just MUFA-containing ones, and caused comparable changes in the acyl chain composition of cellular and effluxed PC molecules (Figs. 2*E*, 3*C* and 8*C*). Thus, the acyl chain composition of the PC molecules effluxed to apoA-I can be determined from that of the cellular PC molecules. Furthermore, these results demonstrated that ABCA1 can transport a variety of cellular PC molecules, including MUFA-containing species, without an apparent preference for their acyl chain structure.

The broad substrate preferences of ABCA1 may explain the diverse functions of ABCA1. ABCA1 is expressed in a wide range of cells and plays various roles under various circumstances. ABCA1 and ABCG1 regulate the proliferation of hematopoietic stem cells (40). In addition, ABCA1 transports cellular lipids to apolipoprotein E in the central nervous system and regulates neuronal structure and functions (41). ABCA1 is also reported to be involved in regulating the migration and social behavior of cells (42, 43). Although ABCA1 functions in a wide range of cell types, cellular phospholipid composition differs depending on the cell type (11). Thus, the fatty acid composition of PC molecules available to ABCA1 may differ depending on the cell type. Although membrane lipid composition influences the expression level of ABCA1, the ability of ABCA1 to transport PC molecules with diverse fatty acyl chains may enable ABCA1 to carry out pleiotropic roles in a variety of cells with diverse phospholipid compositions.

PC(34:1), which is mainly composed of C16:0 and C18:1, is the most abundantly effluxed to apoA-I from ABCA1-expressing HEK293 cells, followed by PC(32:1) and PC(36:2) (17, 44). As the most abundant cellular PC species in HEK293 cells was PC(34:1), followed by PC(32:1) and PC(36:2) (17), the acyl chain composition of PC molecules effluxed to apoA-I from ABCA1-expressing cells likely reflects that of cellular PC even in HEK293 cells. Similar to ABCA1, ABCB4 and ABCG1 transport cellular phospholipids to bile acids and HDL, respectively (44, 45). The acyl chain composition of PC molecules transported to the extracellular taurocholate from ABCB4-expressing HEK293 cells is similar to that of PC molecules effluxed to apoA-I from ABCA1-expressing cells (44, 45). In addition, the composition of PC molecules effluxed from ABCG1-expressing cells is similar to that from ABCA1-expressing cells (44). Thus, these three ABC proteins transported similar PC species when expressed in HEK293 cells. Therefore, it is likely that ABCB4 and ABCG1 also transport PC molecules with diverse fatty acyl chains and do not distinguish differences in the acyl chain structure of PC. However, we cannot exclude the possibility that ABCB4 and ABCG1 preferentially transport PC(34:1) over the other PC species. Therefore, the transport substrate preferences for ABCB4 and ABCG1 need to be verified using the method described in this study. Similar to the role of ABCA1 in HDL production, ABCA3 is involved in surfactant production by transporting phospholipids in alveolar type II cells; thus, ABCA3 deficiency impairs surfactant secretion (46). Since the surfactant is rich in C16:0-containing species (46), ABCA3 may selectively transport C16:0-containing phospholipids during surfactant production; however, the substrate preference of ABCA3 is not fully understood. In addition, ABCA7 also has the activity of transporting cellular phospholipids to the extracellular apoA-I (47), and polymorphisms in the *ABCA7* gene are associated with risk of Alzheimer’s disease (48). Our approach (*i.e.*, examining phospholipid molecules transported from cells with altered phospholipid compositions) provides insights into the transport mechanisms of ABC proteins that serve diverse physiological functions by transporting cellular phospholipids.

The fatty acid composition of PC molecules in circulating HDL particles changes owing to various factors, including food intake and disease progression (49, 50, 51). For example, eicosapentaenoic acid (C20:5) and C22:6 contents in the phospholipid acyl chains of HDL particles are negatively correlated with the severity of coronary artery disease (51). Because the fatty acid composition of HDL phospholipids affects the physicochemical properties and cholesterol-accepting activity of HDL (52), PUFAs within the phospholipid acyl chains of HDL may exert anti-atherosclerotic effects by modulating the function of HDL particles. However, considering our findings, it is likely that the PUFA content in HDL phospholipids does not solely reflect changes in the function of HDL particles. We showed that the fatty acid composition of PC molecules in HDL particles could be influenced by that in HDL-producing cells. Hepatic ABCA1 is a determining factor for circulating HDL levels (53). Thus, although HDL remodeling during circulation and lipid efflux from extrahepatic cells may also be involved (54), the PUFA content in HDL phospholipids may reflect the content of hepatic PUFAs, which contribute to maintaining lipid homeostasis by controlling lipoprotein production and the expression of lipid metabolism-related genes (55). Therefore, our findings provide a molecular basis for the PUFA content in HDL phospholipids as a meaningful indicator of the progression and risk of coronary artery disease.

Previous reports have shown that excess MUFAs decrease the expression level of ABCA1 by enhancing the degradation of ABCA1 protein (19, 20). In this study, we found that reducing MUFA content by SCD1 inhibition suppressed the functional expression of ABCA1. When MUFA content was decreased by SCD1 inhibition, ER stress increased, resulting in a decrease in the production of ABCA1 protein (Figs. 4, *A*–*C* and 5, *A*–*E*). Furthermore, decreased MUFA content affected complex-type glycosylation and plasma membrane localization of ABCA1 (Fig. 4, *D*–*F*). The chemical chaperone 4-PBA rescued the defects in glycosylation and localization of ABCA1 in SCD1-inhibited cells (Fig. 6, *B* and *C*), demonstrating that these defects were caused by folding defects in the ABCA1 protein. However, because the decreased expression of ABCA1 and the increased expression of CHOP in SCD1-inhibited cells were not ameliorated by 4-PBA treatment (Fig. 6, *A* and *D*), the induction of the ER stress response that suppresses ABCA1 protein production in SCD1 inhibitor-treated cells was not caused by a folding defect of proteins that can be rescued by the chemical chaperone. Furthermore, because the ER stress response induced by thapsigargin treatment did not affect the glycosylation and localization of ABCA1 (Fig. 5, *F* and *G*), the defects in glycosylation and localization of ABCA1 upon SCD1 inhibition were not caused by the ER stress response. Thus, these results reveal that reducing the MUFA content in the acyl chains of cellular phospholipids suppresses the functional expression of ABCA1 through two independent mechanisms: first, by inducing an ER stress response that decreases ABCA1 protein production; and second, by causing a folding defect in the ABCA1 protein, leading to immature glycosylation and failure of ABCA1 localization to the plasma membrane. Consistent with previous reports (19, 20), an excess supply of C18:1 decreased ABCA1 expression by enhancing ABCA1 degradation (Fig. 7, *B* and *C*). In contrast to the effect of SCD1 inhibition, an excess supply of C18:1 did not cause ER stress or defects in the glycosylation or localization of ABCA1 (Figs. 7, *D*, *E* and S6). Therefore, the excess supply of MUFA and the reduction in cellular MUFAs lead to a common outcome: a decrease in the functional expression of ABCA1 through different mechanisms.

Interactions with specific membrane lipids affect membrane protein oligomerization (56), and the dynamic regulation of ABCA1 oligomerization during HDL production has been proposed (57, 58, 59). Since a decrease in the MUFA content in phospholipid acyl chains affects the structure of ABCA1, exploring how the MUFA content in phospholipids influences the higher-order structure of ABCA1 is an intriguing topic. However, the contribution of membrane lipids to ABCA1 oligomerization remains controversial, and it has been suggested that membrane lipids are not required for ABCA1 oligomerization (57), whereas other studies have suggested that the lipid transport activity of ABCA1 promotes ABCA1 dimerization (59). Investigating how changes in the fatty acid composition of cellular phospholipids regulate the higher-order structure of ABCA1 will deepen our understanding of the mechanisms underlying HDL formation by ABCA1.

In summary, we elucidated the effect of the acyl chain structure of cellular phospholipids on ABCA1-mediated HDL formation, with a particular focus on MUFA-containing species. PC molecules containing one or two MUFAs accounted for 58% and 25% of the total cellular PC molecules, respectively (Fig. 1*C*), suggesting that the ABCA1 protein is surrounded by numerous PC molecules containing MUFAs. MUFA-containing PC was abundant in the HDL particles produced; however, MUFA-containing PC was not preferentially transported to apoA-I over other PC species by ABCA1. In contrast, an appropriate amount of MUFA-containing phospholipids is required for the functional expression of ABCA1. Reducing the MUFA content in the acyl chains of cellular phospholipids causes a decrease in the functional expression of ABCA1 through the ER stress response and folding defect of ABCA1 protein, whereas increasing the MUFA content by excess supply reduces the expression level of ABCA1 through a mechanism distinct from that observed in MUFA depletion. Thus, we uncovered the contribution of MUFA-containing PC to HDL formation and identified the structural characteristics of the cellular phospholipids required for their transport to apoA-I and the functional expression of ABCA1. Furthermore, our study revealed that appropriate regulation of the acyl chain composition of cellular phospholipids is important for ABCA1-mediated HDL formation and that the dysregulation of cellular phospholipid composition may affect the production and function of HDL particles.

## Experimental procedures

### Materials and cell culture

BHK/ABCA1 cells (60), in which the expression of human ABCA1 could be induced by the addition of mifepristone, and Raw264 cells were grown in a humidified incubator (5% CO_2_) at 37 °C in Dulbecco's modified Eagle's medium (DMEM) supplemented with 10% heat-inactivated fetal bovine serum (FBS) (BioWest). ABCA1 expression in BHK/ABCA1 cells was induced by incubation for 24 h with 10 nM mifepristone (Sigma-Aldrich) in DMEM containing 0.02% bovine serum albumin (BSA). ABCA1 expression in Raw264 cells was induced by incubation for 24 h with 10 μM TO901317 (Cayman Chemical) and 5 μM 9cis-retinoic acid (Sigma-Aldrich). CAY10566 (Cayman Chemical), thapsigargin (Sigma), 4-PBA (Wako), and cycloheximide (Wako) were added to the culture medium at concentrations of 1 μM, 1 μM, 10 mM, and 100 μg/ml, respectively. Free fatty acids (C18:1, C20:4, and C22:6) were purchased from Sigma-Aldrich. The BSA-fatty acid complex (molar ratio of 1:9) was prepared by incubating the fatty acids in a 0.9% NaCl solution containing BSA (111 mg/ml). Cells were incubated with the BSA-fatty acid complex for 24 h to increase the cellular content of specific fatty acid species. The coding sequence of Flag-tagged mouse SCD1 (38) was ligated into multiple cloning sites of the pcDNA3.1(+) vector to construct pcDNA3.1-SCD1-Flag. BHK/ABCA1 cells were transfected with pcDNA3.1-SCD1-Flag using Lipofectamine 3000 Reagent (Thermo Fisher Scientific). Human apoA-I was expressed as a thioredoxin fusion protein in *Escherichia coli* strain BL21-DE3 host, and then cleaved and purified as described previously (61).

### Measurement of fatty acid composition of phospholipids by gas chromatography (GC)

Total lipids were extracted using the Bligh and Dyer procedure (62). Phospholipids were separated by thin-layer chromatography (TLC) using hexane/diethyl ether/acetic acid (60:40:1, v/v/v) and incubated with methanolic HCl at 100 °C for 3 h. Fatty acid methyl esters were extracted and analyzed using a Shimadzu GC-2014 with a flame ionization detector (FID) and an Omegawax Capillary GC column (Supelco). The temperature of the injector and the flame ionization detector were held at 200 and 280 °C, respectively. The column temperature was programmed as follows: held at 180 °C for 5 min; ramped to 220 °C at 3 °C/min; held for 7 min; ramped to 240 °C at 3 °C/min; and held for 10 min. The peak areas of methyl esters of C14:0, C16:0, C16:1, C18:0, C18:1, C18:2, C20:4, and C22:6 were determined.

### Liquid chromatography–mass spectroscopy analysis of phospholipids

Phospholipids were analyzed using a Shimadzu LC-30AD high-performance liquid chromatography system coupled with a triple-quadrupole LCMS-8040 mass spectrometer equipped with an electrospray source (63). Separation was performed on a Kinetex C8 column (2.6 μm; 2.1 × 150 mm; Phenomenex) with a binary mobile phase of the following composition: 10 mM ammonium formate in water (mobile phase A) and 10 mM ammonium formate in 2-propanol/acetonitrile/water (45:45:10; v/v/v) (mobile phase B). The pump controlling the mobile phase B gradient was programmed as follows: an initial isocratic flow of 20% B for 1 min, a linear increase to 40% B for 1 min, an increase to 92.5% B using a curved gradient for 23 min, a linear increase to 100% B for 1 min, and a hold at 100% B for 4 min. The total flow rate was 0.3 ml/min, the column temperature was 45 °C, and the sample temperature was 4 °C. The spectrometer parameters were as follows: nebulizer gas flow, 2 L/min; drying gas flow, 15 L/min; interface voltage, 4.5 kV; DL temperature, 250 °C; and heat-block temperature, 400 °C. The multiple reaction monitoring transition for PC was [M + H]^+^/[184.1]^+^. The fatty acid composition of PC was determined by the product ion scan analysis of [M + HCOO]^-^ as a precursor ion.

### Cholesterol and phospholipid efflux

Cells were incubated for 24 h with 10 nM mifepristone in DMEM containing 0.02% BSA in the presence or absence of inhibitors, and then incubated for 4 h with 10 μg/ml apoA-I in DMEM containing 0.02% BSA in the presence or absence of inhibitors. The medium was collected, and the remaining cell monolayers were lysed in 0.1 N NaOH. The protein concentration of the lysed cells was determined using a BCA assay. The cholesterol content in the medium was determined using a fluorescent enzyme assay (64). The PC content in the medium was analyzed by LC-ESI-MS using PC(25:0) as the internal standard (IS).

### Cellular cholesterol and triacylglycerol measurement

Cellular cholesterol content was determined using a fluorescent enzyme assay (64). The cellular triacylglycerol content was measured using a triglyceride assay kit (Wako).

### Immunoblotting

The cells were washed with PBS and lysed in lysis buffer (10 mM Tris-HCl, pH 7.4, 1% Triton X-100, 0.1% SDS, and 1% sodium deoxycholate) containing 1% protease inhibitor cocktail (Nacalai Tesque). The lysates were centrifuged at 14,000×*g* for 10 min at 4 °C. 20 μl of supernatant was mixed with 5 μl of sampling buffer 1 (50% sucrose, 50 mM Tris-HCl, pH 8.0, 1% SDS, 5 mM EDTA, 0.4% bromophenol blue), incubated for 10 min at 50 °C, and then mixed with 25 μl of sampling buffer 2 (10% sucrose, 10 mM Tris-HCl, pH 8.0, 0.2% SDS, 1 mM EDTA, 0.08% bromophenol blue, 60% urea). Samples were electrophoresed on an SDS-polyacrylamide gel and blotted onto a PVDF membrane (Wako) using a Trans-Blot SD Semi-Dry Electrophoretic Transfer Cell (Bio-Rad). Immunoblotting analysis was performed by using anti-ABCA1 antibody (Santa Cruz, sc-58219), anti-CHOP antibody (Proteintech, 15204-1-AP), anti-LDLR antibody (Proteintech, 10785-1-AP), and anti-α-tubulin antibody (MBL, PM054) as a loading control. Bound antibodies were detected with horseradish peroxidase-conjugated anti-rabbit IgG and anti-mouse IgG antibodies using Super Signal West Pico (Thermo Fisher Scientific) and LuminoGraph I (Atto). Band intensity was determined using ImageJ software.

### Glycosylation analysis

Endo H and PNGase F (New England Biolabs) digestions were performed as described by the manufacturer. In brief, 5 μl of cell lysate was treated with 250 units of Endo H or 250 units of PNGaseF for 1 h at 37 °C. Deglycosylated proteins were separated using SDS-polyacrylamide gel electrophoresis and analyzed by immunoblotting.

### Real-time PCR

Total RNA was extracted using NucloSpin RNA (Takara). cDNA was prepared using the ReverTra Ace qPCR RT Master Mix (Toyobo). The expression level of *ABCA1* mRNA was quantified using StepOne real-time PCR system (Applied Biosystems) with PowerUP SYBR Green Master Mix (Thermo Fisher Scientific) and specific primers (for ABCA1, 5′- CAGGCTACTACCTGACCTTGGT -3′ and 5′- CTGCTCTGAGAAACACTGTCCTC -3′; for GAPDH, 5′- GCACAGTCAAGGCTGAGAA -3′ and 5′- GCCAGTAGACTCCACAACATAC -3′) and quantified using the 2^-ΔΔCt^ method.

### Biotinylation of cell surface proteins

The cells were washed with ice-cold PBS and incubated with 0.5 mg/ml sulfo-NHS-biotin solubilized in PBS for 30 min on ice. Cells were washed with TBS (20 mM Tris-Cl, pH 7.4, 150 mM NaCl) to remove unbound sulfo-NHS-biotin and lysed in lysis buffer (20 mM Tris-Cl, pH 7.5, 1% Triton X-100, 0.1% SDS, and 1% sodium deoxycholate) containing protease inhibitors. An immobilized monomeric avidin gel (Pierce) was added to the cell lysates to precipitate the biotinylated proteins, which were electrophoresed on a 7.5% SDS-polyacrylamide gel and immunodetected.

### Cell viability assay

Cell viability was estimated by measuring the LDH activity in the medium and cells using the Cytotoxicity LDH Assay Kit-WST (Dojindo).

### Statistical analysis

Values are presented as means ± SD. The numbers of biological replicates (*n*) are indicated in the figure legends. The statistical significance of the differences between mean values was analyzed using a non-paired *t* test. Multiple comparisons were performed using Tukey’s test followed by analysis of variance. Statistical significance was set at *p* < 0.05. The experiments were conducted multiple times, and consistent results were obtained.

## Data availability

All data are contained within the article and supporting information.

## Supporting information

This article contains supporting information.

## Conflict of interest

The authors declare that they have no conflicts of interest with the contents of this article.
