# Supplementary material for: The structural characteristics of cellular phospholipid acyl chains required for ABCA1-mediated HDL formation
Source: J Biol Chem. 2025 Jul 4;301(8):110457. doi: 10.1016/j.jbc.2025.110457 (PMC12336828; doi:10.1016/j.jbc.2025.110457)
Supplement: Supplementary Information [file mmc1.pdf]

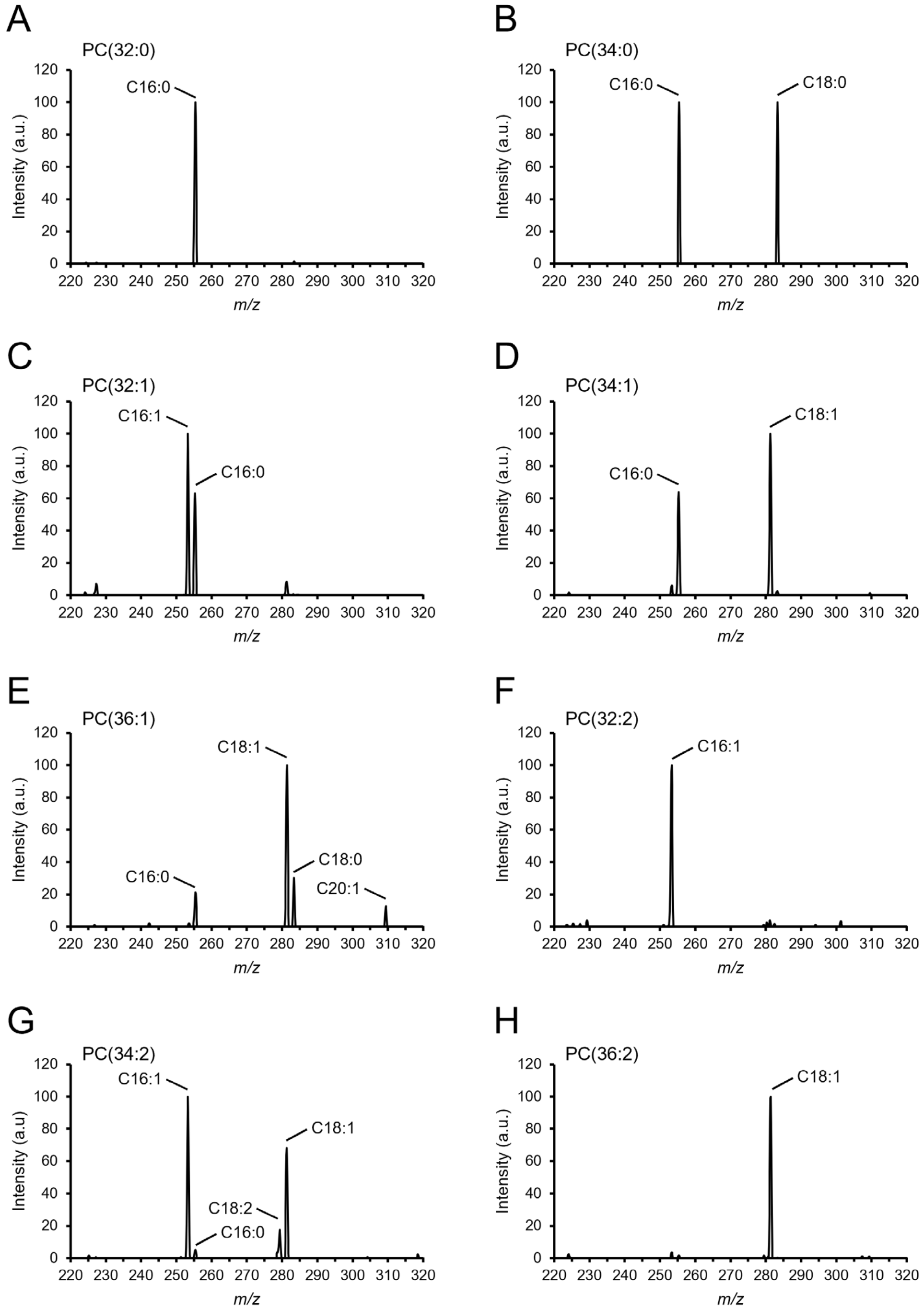

Supplemental Fig. S1. Fatty acid composition of PC molecules in BHK/ABCA1 cells. BHK/ABCA1 cells were treated with 10 nM mifepristone for 24 h. Product ion scan analysis of PC(32:0) (A), PC(34:0) (B), PC(32:1) (C), PC(34:1) (D), PC(36:1) (E), PC(32:2) (F), PC(34:2) (G), and PC(36:2) (H) extracted from BHK/ABCA1 cells.

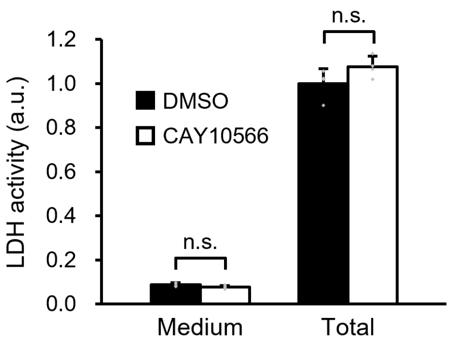

Supplemental Fig. S2. Effect of SCD1 inhibition on cell viability. BHK/ABCA1 cells were treated with 10 nM mifepristone in the presence or absence of 1 μM CAY10566 for 24 h. The lactate dehydrogenase (LDH) activity in the medium and cells was analyzed. Total represents LDH activity in medium + cells. Mean ± SD (n = 4). n.s., not significant; a.u., arbitrary unit.

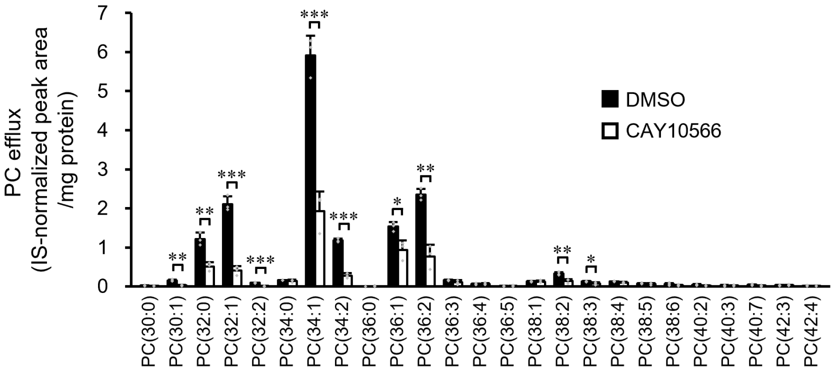

Supplemental Fig. S3. Effect of SCD1 inhibition on the amount of effluxed PC molecules. BHK/ABCA1 cells were treated with 10 nM mifepristone in the presence or absence of 1 μM CAY10566 for 24 h, and then incubated with 10 μg/mL apoA-I for 4 h. The amount of PC molecules effluxed to apoA-I was analyzed by LC-ESI-MS. The amount of effluxed PC molecules were normalized with the peak area of internal standard (IS) and the cellular protein content. PC molecules were presented in the format PC(X:Y), where X denotes the total number of acyl chain carbons and Y denotes the total number of double bonds in acyl chains. Mean ± SD (n = 3). *$P < 0.05$; **$P < 0.01$; ***$P < 0.001$.

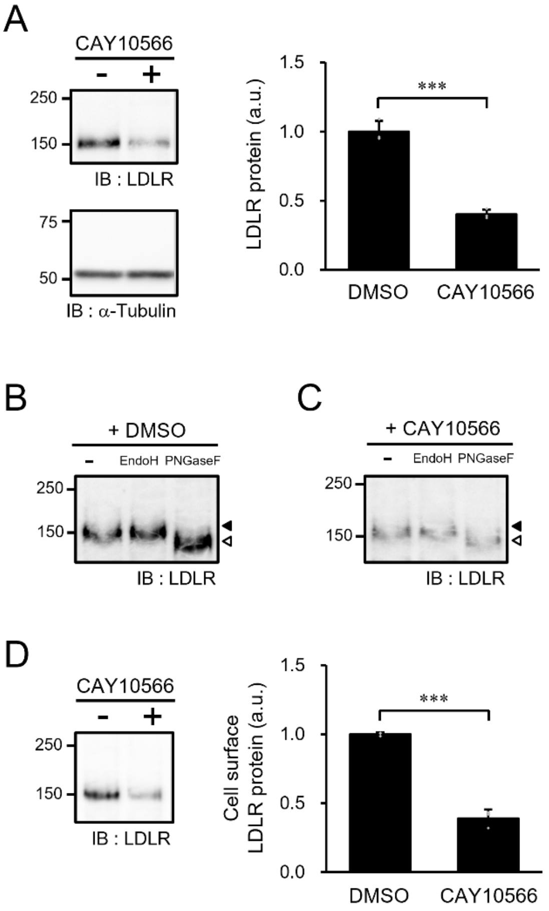

Supplemental Fig. S4. Effect of SCD1 inhibition on the expression of LDLR. BHK/ABCA1 cells were treated with 10 nM mifepristone in the presence or absence of 1 μM CAY10566 for 24 h. (A) The amounts of LDLR and α-tubulin protein were detected with specific antibodies. Glycosylation analysis of LDLR in BHK/ABCA1 cells treated with DMSO (B) and 1 μM CAY10566 (C). Closed arrowhead indicates the position of LDLR in sample not treated with glycosidases; open arrowhead indicates the position of deglycosylated LDLR. (D) The cells were treated with sulfo-NHS-biotin, and the biotinylated cell surface proteins were precipitated with avidin-agarose and detected by immunoblotting. (A-D) Numbers on the left of the panels indicate the molecular weights (kDa) of size markers. Mean ± SD (A, D, n = 3). ***$P < 0.001$; a.u., arbitrary unit.

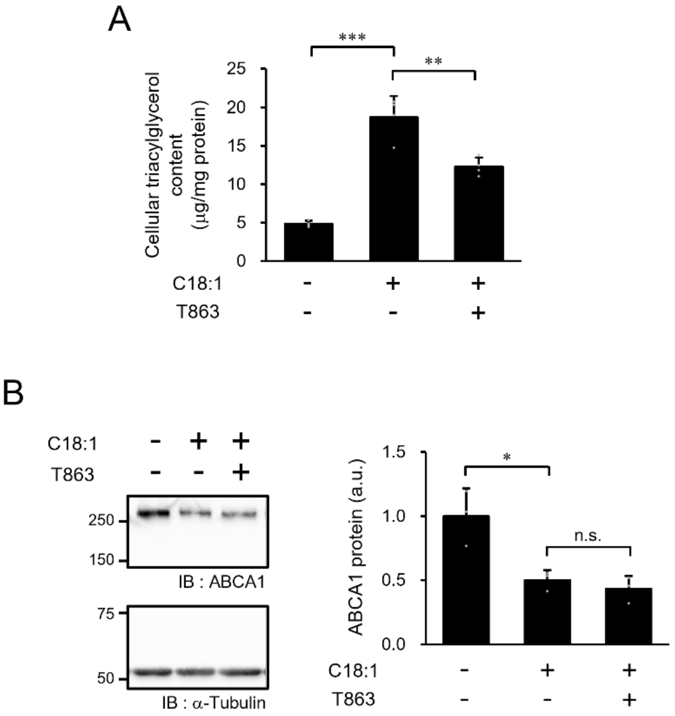

Supplemental Fig. S5. Effect of excess MUFA on triacylglycerol production. BHK/ABCA1 cells were treated with 10 nM mifepristone in the presence or absence of 125 μM C18:1 and 10 μM T863 for 24 h. (A) Cellular triacylglycerol content was analyzed by a colorimetric enzyme assay. (B) The amounts of ABCA1 and α-tubulin protein were detected with specific antibodies. Numbers on the left of the panels indicate the molecular weights (kDa) of size markers. Mean ± SD (A, B, n = 3). *$P < 0.05$; **$P < 0.01$; ***$P < 0.001$; n.s., not significant; a.u., arbitrary unit.

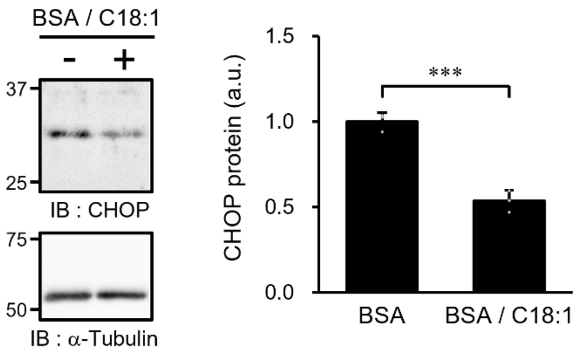

Supplemental Fig. S6. Effect of excess MUFA on ER stress response. BHK/ABCA1 cells were treated with 10 nM mifepristone in the presence or absence of 125 μM C18:1 for 24 h. The amounts of CHOP and α-tubulin protein were detected with specific antibodies. Numbers on the left of the panels indicate the molecular weights (kDa) of size markers. Mean ± SD (*n* = 3). ****P* < 0.001; a.u., arbitrary unit.

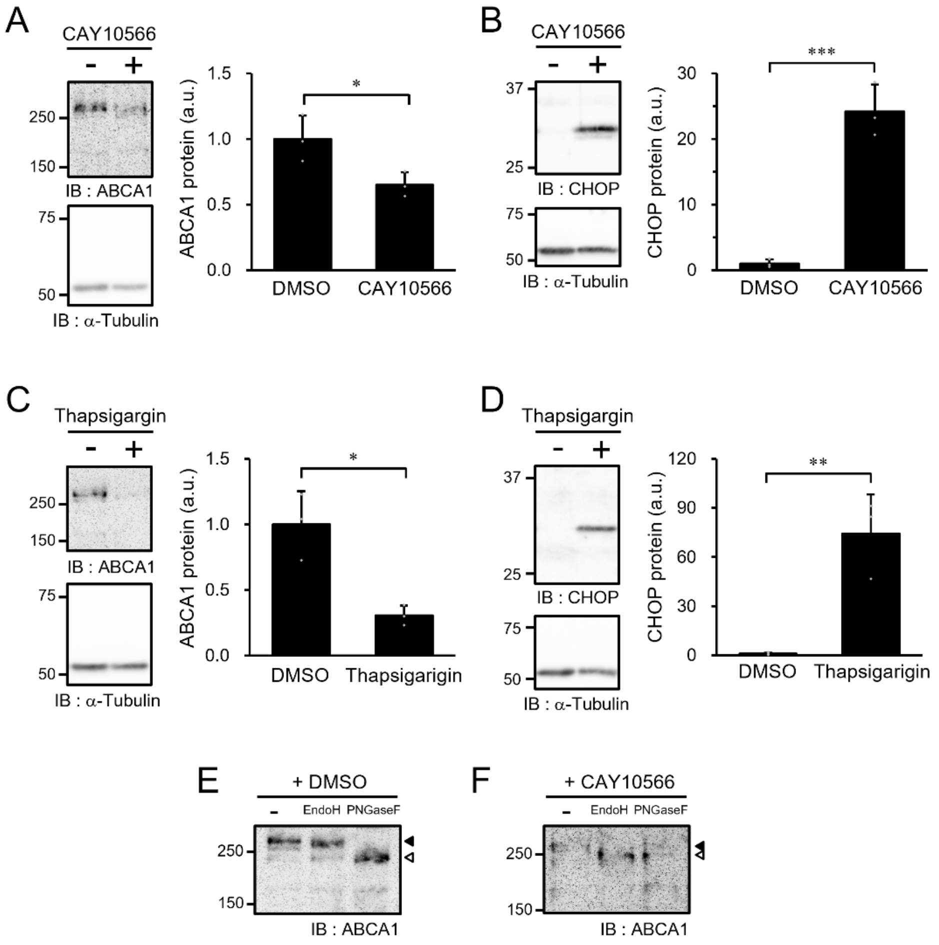

Supplemental Fig. S7. Effect of SCD1 inhibition on the expression of ABCA1 in Raw264 cells. Raw264 cells were treated with 10 μM TO901317 and 5 μM 9cis-retinoic acid in the presence or absence of 1 μM CAY10566 or 1 μM thapsigargin for 24 h. (A-D) The amounts of ABCA1, CHOP, and α-tubulin protein were detected with specific antibodies. Glycosylation analysis of ABCA1 in Raw264 cells treated with DMSO (E) and 1 μM CAY10566 (F). Closed arrowhead indicates the position of ABCA1 in sample not treated with glycosidases; open arrowhead indicates the position of deglycosylated ABCA1. (A-F) Numbers on the left of the panels indicate the molecular weights (kDa) of size markers. Mean ± SD (A-D, $n = 3$). *$P < 0.05$; **$P < 0.01$; ***$P < 0.01$; a.u., arbitrary unit.

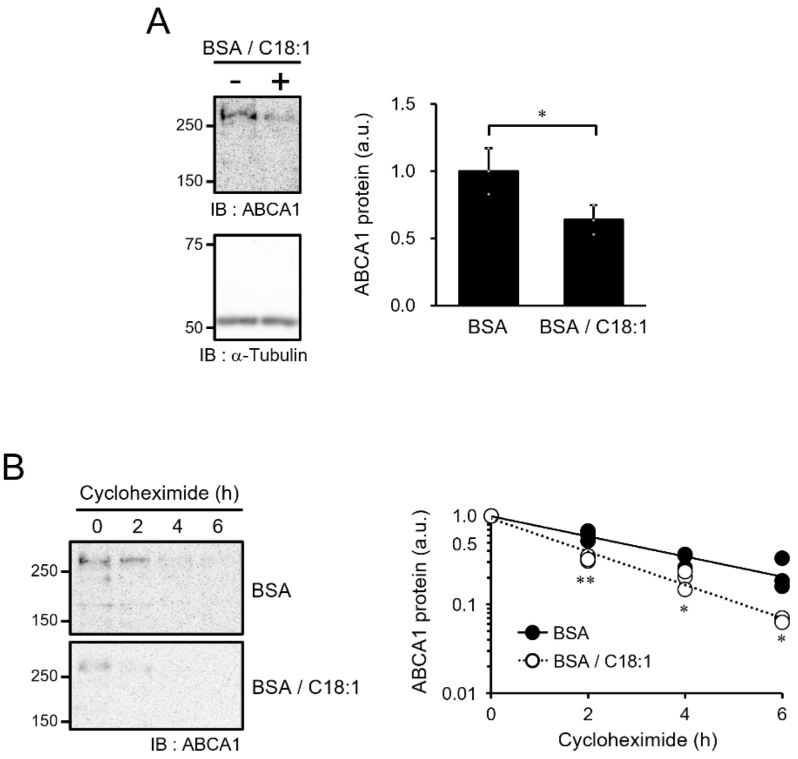

Supplemental Fig. S8. Effect of excess MUFA on the expression of ABCA1 in Raw264 cells. Raw264 cells were treated with 10 μM TO901317 and 5 μM 9cis-retinoic acid in the presence or absence of 125 μM C18:1 for 24 h. (A) The amounts of ABCA1 and α-tubulin protein were detected with specific antibodies. (B) Cells were treated with cycloheximide (100 μg/mL) for 0, 2, 4, and 6 h in the presence or absence of 125 μM C18:1, and the amounts of ABCA1 protein were detected with specific antibodies (n = 3). (A, B) Numbers on the left of the panels indicate the molecular weights (kDa) of size markers. Mean ± SD (A, n = 3). $*P < 0.05$; $**P < 0.01$; a.u., arbitrary unit.

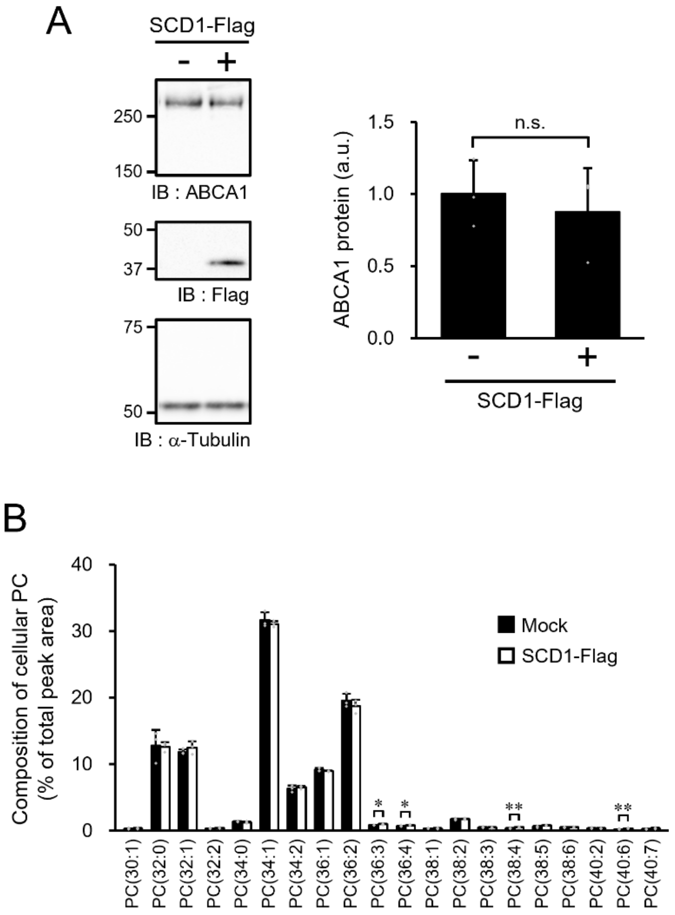

Supplemental Fig. S9. Effect of SCD1 expression on the expression of ABCA1. BHK/ABCA1 cells were transfected with or without SCD1-Flag-expression plasmid and treated with 10 nM mifepristone for 24 h. (A) The amounts of ABCA1, SCD1-Flag, and α-tubulin protein were detected with specific antibodies. Numbers on the left of the panels indicate the molecular weights (kDa) of size markers. (B) Composition of cellular PC molecules was analyzed by LC-ESI-MS. PC molecules were presented in the format PC(X:Y), where X denotes the total number of acyl chain carbons and Y denotes the total number of double bonds in acyl chains. Mean ± SD (A, B, *n* = 3). **P* < 0.05; ***P* < 0.01; n.s., not significant; a.u., arbitrary unit.
